# Knowledge and Psychological Stress Related to COVID-19 Among Nursing Staff in a Hospital in China: Cross-Sectional Survey Study

**DOI:** 10.2196/20606

**Published:** 2020-09-18

**Authors:** Huaping Huang, Wen-Jun Zhao, Gui-Rong Li

**Affiliations:** 1 Operating Room Mianyang Central Hospital Mianyang China; 2 Department of Nursing Mianyang Central Hospital Mianyang China

**Keywords:** COVID-19, nursing staff, knowledge, psychological stress

## Abstract

**Background:**

Since December 2019, coronavirus disease (COVID-19) has been rapidly spreading worldwide. Nurses play a key role in fighting this disease and are at risk of COVID-19 infection. Therefore, there is an urgent need to assess the mental health condition of nurses and establish appropriate interventions to reduce the negative psychiatric outcomes of the pandemic.

**Objective:**

The objectives of this study were to evaluate the knowledge and psychological stress related to COVID-19 among nursing staff and to provide evidence of the need for targeted training and psychological intervention.

**Methods:**

This cross-sectional web-based survey study was performed in a class 3 grade A general hospital in a southwest province of China from March 1 to March 15, 2020. A self-designed questionnaire with questions about COVID-19–related prevention and control knowledge and the Triage Assessment Form (TAF) were used to assess nursing staff’s knowledge of COVID-19 and their degree of psychological stress, respectively. SPSS 23.0 was applied for statistical analysis of the collected data.

**Results:**

A total of 979 nurses completed the questionnaire. The results showed that the nursing staff provided the fewest correct answers to questions about continuous viral nucleic acid testing specifications (379/979 correct answers, 38.7%), isolation/discharge criteria (539/979 correct answers, 55.1%), and management measures for patients with suspected symptoms (713/979 correct answers, 72.8%). The median total score of the TAF was 7.0 (IQR 5.0-12.0), and there were statistically significant differences in scores between different nursing roles, years of work experience, and hospital departments (*P*<.05).

**Conclusions:**

This study indicated that nursing staff have insufficient knowledge about COVID-19. Meanwhile, although the psychological damage to nurses during the pandemic was found to be low, nurse managers must continue to monitor the mental health of nursing staff and perform timely interventions.

## Introduction

Since December 2019, increasing numbers of new cases of coronavirus disease (COVID-19) have emerged in Wuhan City, Hubei Province [1]. New cases have also spread in other cities in China as well as in other countries worldwide. In China, the disease has been classified as a Class B infectious disease that requires the same management as a Class A infectious disease [2]. Gene sequencing analysis revealed that the virus is highly homologous to severe acute respiratory syndrome coronavirus (SARS-CoV) found in bats [3]. The incubation period of COVID-19 can be 1 to 14 days but is usually 3 to 7 days. The main route of transmission is through interpersonal respiratory droplets or contact [4]. Early detection, early diagnosis, early isolation, and early treatment have become the most effective measures to limit the transmission of COVID-19. For nurses who are fighting the pandemic on the front line, knowledge about COVID-19 prevention will be important to adopt precise management strategies. Meanwhile, heavy workload, shortage of personal protective equipment, and inadequate support may contribute to different levels of psychological stress [5]. Therefore, the objective of this study is to evaluate nursing staff’s knowledge about COVID-19 and their psychological stress.

## Methods

### Study Design and Participants

This is a cross-sectional survey study. Nurses who had participated in the prevention and control of COVID-19 and were willing to take part in this survey were recruited from a class 3 grade A comprehensive hospital in a southwest province of China from March 1 to 15, 2020. This study was reviewed and approved by the ethics committee of the hospital (reference number: 2020-03), and informed consent was obtained from all participants.

### Questionnaire

The questionnaire included 3 parts: general information, knowledge about the prevention and control of COVID-19, and the Triage Assessment Form (TAF) for psychological crisis intervention [6]. General information included age, gender, education, job title, personnel category, and department. The questions regarding knowledge about the prevention and control of COVID-19 were mainly designed based on the *Prevention and Control Plan for COVID-19* [7] issued by the National Health Commission of China; this part consisted of 10 items, 2 of which were multiple-choice questions related to the nurses’ amount of patient contact and sources of information about COVID-19 and 8 of which were true or false questions regarding prevention and control strategies for COVID-19. The TAF included three dimensions: emotion, behavior, and knowledge. Each dimension used a 10-point scoring method; “never” scored 1 point and “always” scored 10 points, with a total score of 3 to 30 points. The higher the score, the more severe the mental health damage. The Cronbach α value of the scale was .81.

### Survey Distribution

To abide by the principle of informed consent, a unified guideline was used to inform participants of the purpose of this study and its confidentiality measures. A web-based survey (via a questionnaire website platform) was sent to the head nurse of each ward, who was asked to send the survey to other nurses. Participants could complete the questionnaire using a computer or a smartphone that could open a website link or scan a Quick Response code.

### Statistical Analysis

SPSS 23.0 (IBM Corporation) was used to analyze the collected data. The binary variables were expressed in the form of percentages. For continuous variables, if they conformed to the normal distribution, the results are presented as mean (SD). Otherwise, they are presented as median (IQR). Univariate analysis was performed using the Wilcoxon rank test or Kruskal-Wallis test. *P*<.05 was considered to indicate a statistically significant difference.

## Results

### Demographic Characteristics of the Participants

A total of 981 questionnaires were collected in this survey, of which 2 questionnaires were rejected due to imperfect information. Finally, 979 valid questionnaires were obtained, with a questionnaire effectiveness rate of 99.8%. As shown in Table 1, the majority of the nursing staff who participated in the survey were in-service nurses who had a bachelor’s degree, held a junior professional title, and had less than 5 years of working experience; the average age of the participants was 29.68 years (SD 7.35).

**Table 1:** Demographic characteristics of the study participants (N=979).

Characteristic		Value
**Age (years), mean (SD)**		29.68 (7.4)
	≤30	662 (67.6)
	>30	317 (32.4)
**Gender, n (%)**		
	Male	45 (4.6)
	Female	934 (95.4)
**Personnel category, n (%)**		
	Student nurse	113 (11.5)
	Trainee nurse	118 (12.1)
	In-service nurse	748 (76.4)
**Education** **level, n (%)**		
	Technical secondary school	4 (0.4)
	Junior college	301 (30.8)
	Undergraduate college	668 (68.2)
	Postgraduate college	6 (0.6)
**Professional title, n (%)**		
	Nurse practitioner	674 (68.9)
	Supervisor nurse	247 (25.2)
	Co-chief operator nurse	55 (5.6)
	Chief operator nurse	3 (0.3)
**Work experience (years)**		
	<1	203 (20.7)
	1-5	263 (26.9)
	6-10	284 (29.0)
	10-15	92 (9.4)
	16-20	47 (4.8)
	>20	90 (9.2)
**Department**		
	Emergency	47 (4.8)
	Outpatient	51 (5.2)
	Respiratory	54 (5.5)
	Critical care medicine	48 (4.9)
	Infection	9 (0.9)
	General ward	437 (44.6)
	Other	333 (34.0)

**TAF Scores Among Nursing Staff**

The median total TAF score of the nursing staff was 7.0 (IQR 5.0-12.0), indicating that the psychological damage suffered by the nursing staff was slight. There were statistically significant differences in the total TAF score and the score of each domain between the different personnel categories, years of work experience, and departments (*P*<.05; Table 2).

**Table 2:** Status of psychological stress among nursing staff (N=979) based on their Triage Assessment Form scores, median (IQR).

Characteristic		Emotion	Knowledge	Behavior	Total
**Age (years)**					
	≤30	3.0 (2.0-4.0)	2.0 (1.0-4.0)	2.0 (1.0-4.0)	8.0 (5.0-11.3)
	＞30	3.0 (2.0-5.0)	2.0 (1.0-4.0)	2.0 (1.0-4.0)	7.0 (4.0-13.0)
	*z* value	–1.387	–0.046	–1.080	–0.356
	*P* value	.17	.96	.28	.72
**Gender**					
	Male	3.0 (1.0-4.0)	2.0 (1.0-3.0)	2.0 (1.0-4.0)	6.0 (3.5-12.0)
	Female	3.0 (2.0-4.3)	2.0 (1.0-4.0)	2.0 (1.0-4.0)	7.0 (5.0-12.0)
	*z* value	–1.154	–1.240	–0.990	–1.411
	*P* value	.25	.22	.32	.16
**Personnel category**					
	Student nurse	3.0 (2.0-5.0)	4.0 (2.0-5.0)	3.0 (2.0-5.0)	11.0 (6.0-15.0)
	Trainee nurse	3.0 (2.0-4.0)	2.0 (1.0-3.0)	2.0 (1.0-3.0)	7.5 (5.0-11.0)
	In-service nurse	3.0 (2.0-4.0)	2.0 (1.0-4.0)	2.0 (1.0-3.0)	7.0 (5.0-12.0)
	*H* value	8.613	24.576	25.796	17.433
	*P* value	.01	<.001	<.001	<.001
**Education**					
	Technical secondary school	2.5 (1.3-3.0)	3.0 (2.3-3.8)	3.0 (1.5-3.8)	8.0 (5.5-10.5)
	Junior college	3.0 (2.0-4.5)	2.0 (1.0-4.0)	2.0 (1.0-4.0)	8.0 (5.0-12.0)
	Undergraduate college	3.0 (2.0-4.0)	2.0 (1.0-4.0)	2.0 (1.0-4.0)	7.0 (5.0-12.0)
	Postgraduate college	4.0 (2.8-5.5)	3.5 (2.5-5.0)	3.0 (2.0-5.0)	10.0 (8.0-15.5)
	*H* value	2.857	2.180	2.961	1.773
	*P* value	.41	.54	.40	.62
**Professional title**					
	Nurse practitioner	3.0 (2.0-4.0)	2.0 (1.0-4.0)	2.0 (1.0-3.0)	7.0 (5.0-11.0)
	Supervisor nurse	3.0 (2.0-4.0)	2.0 (1.0-4.0)	2.0 (1.0-4.0)	7.0 (4.0-13.0)
	Co-chief operator nurse	2.0 (1.0-5.0)	2.0 (1.0-5.0)	2.0 (1.0-4.0)	9.0 (4.0-14.0)
	Chief operator nurse	2.0 (2.0-4.0)	6.0 (4.5-7.5)	2.0 (1.5-4.0)	12.0 (9.5-15.0)
	*H* value	1.748	5.022	0.533	2.296
	*P* value	.63	.17	.91	.51
**Work experience (years)**					
	＜1	3.0 (2.0-5.0)	3.0 (2.0-5.0)	3.0 (2.0-5.0)	9.0 (5.0-13.0)
	1-5	3.0 (2.0-4.0)	2.0 (1.0-3.0)	2.0 (1.0-3.0)	7.0 (5.0-11.0)
	6-10	3.0 (2.0-4.0)	2.0 (1.0-4.0)	2.0 (1.0-3.0)	7.0 (5.0-11.0)
	10-15	3.0 (2.0-4.0)	2.0 (1.0-4.0)	2.0 (1.0-4.0)	6.0 (4.0-12.0)
	16-20	2.0 (1.0-6.0)	2.0 (1.0-4.0)	1.0 (1.0-3.0)	6.0 (4.0-13.0)
	＞20	3.0 (1.8-5.0)	3.0 (1.0-4.3)	2.0 (1.0-4.0)	9.0 (5.0-13.3)
	*H* value	4.377	18.330	18.516	12.738
	*P* value	.50	.002	.003	.03
**Department**					
	Emergency	4.0 (3.0-5.0)	3.0 (2.0-5.0)	3.0 (2.0-5.0)	10.0 (7.0-13.0)
	Outpatient	4.0 (2.0-5.0)	3.0 (2.0-5.0)	3.0 (2.0-5.0)	11.0 (6.0-15.0)
	Respiratory	3.0 (2.0-5.0)	3.0 (1.0-4.0)	3.0 (1.0-4.0)	9.0 (5.8-12.0)
	Critical care medicine	3.0 (2.0-4.0)	2.0 (1.0-3.0)	2.0 (1.0-3.0)	7.0 (4.0-9.8)
	Infection	4.0 (2.5-5.5)	4.0 (2.0-6.0)	3.0 (1.5-6.0)	11.0 (6.5-17.0)
	General ward	2.0 (2.0-4.0)	2.0 (1.0-3.0)	2.0 (1.0-3.0)	6.0 (4.0-11.0)
	Other	3.0 (2.0-5.0)	2.0 (1.0-4.0)	2.0 (1.0-4.0)	8.0 (5.0-12.0)
	*H* value	18.734	16.692	14.376	18.042
	*P* value	.01	<.001	<.001	<.001

**Status of Nursing Staff Knowledge About COVID-19**

In this survey, 34 of the 979 nurses (3.5%) nurses reported that they had a very high degree of contact with patients, 214 (21.9%) reported that they had a high degree of contact with patients, and 731 (74%) reported that they had a moderate degree of contact due to indirect contact with patients. The most common approaches by which the nursing staff accessed relevant knowledge were WeChat, Weibo, and official websites. The nursing staff’s knowledge about the prevention and control of COVID-19 reflected that they did not have adequate knowledge of the standards for consecutive viral nucleic acid tests (correct answer rate 379/979, 38.7%), the criteria for release of isolation/discharge (539/979, 55.1%), and the management measures for patients with suspectable symptoms (713/979, 72.8%) (Table 3).

**Table 3:** Knowledge about prevention and control of COVID-19 among nurses (N=979).

Question	Correct answers, n (%)	Ranking
For people who have been exposed to suspicious cases, but do not have any discomfort at present, how does one deal with them subsequently?	947 (96.7)	1
The first symptom of COVID-19?	915 (93.5)	2
Clinical classification of patients with COVID-19?	906 (92.5)	3
Definition of confirmed cases of COVID-19?	905 (92.4)	4
Which mask has the best protective effect when worn correctly?	793 (81.0)	5
Management measures for occurrence of headache, runny nose, cough, sore throat, and other symptoms?	713 (72.8)	6
What are the criteria for patients to be discharged and released from isolation?	539 (55.1)	7
Sampling time interval between the two consecutive respiratory tract nucleic acid tests?	379 (38.7)	8

## Discussion

### Principal Findings

Since the early reports of COVID-19 cases in China in late December 2019, the worst pandemic in 100 years has spread worldwide, with approximately 823,626 confirmed cases and over 40,598 deaths as of April 1, 2020 [8]. The World Health Organization has declared the global COVID-19 outbreak to be a public health emergency of international concern [9]. Due to the atypical pneumonia symptoms and long incubation period of COVID-19 as well as its transmission through respiratory droplets, secretions, and contact, prevention and control of the disease are difficult. Nursing staff are fighting the pandemic on the front lines, and knowledge about the prevention and control of COVID-19 is critical to enable them to provide better care for patients and to protect themselves.

The results of this study showed that nursing staff mainly acquire COVID-19–relevant knowledge through new media platforms such as WeChat and Weibo, followed by the official websites of health administrative departments and professional medical organizations. In the Internet era, WeChat, Weibo, and other platforms have become the most popular learning approaches for health professionals due to their advantages such as mobility, portability, fast update speed, and substantial amounts of information [10]. WeChat has become the most commonly used interactive communication tool for medical education [11] and patient management [12]. However, the information disseminated on social media is also highly arbitrary, especially regarding the COVID-19 outbreak, and response to the pandemic on social media has been accompanied by an overabundance of information [13]. Nurses should have the ability to distinguish false information and avoid being misled. In terms of knowledge related to the prevention and control of COVID-19, the rates of correctness of nursing staff’s answers to questions about the standards for consecutive pathogen nucleic acid tests, the criteria for release from isolation or discharge, and the management measures for patients with suspectable symptoms were relatively low. Multi-round and multi-sample viral nucleic acid tests are of great significance for supporting etiological diagnosis [14]; however, most nurses were unclear about the time interval required between multiple tests (at least 24 hours). At the same time, because the criteria for release from isolation or discharge of patients are mainly determined by doctors, the enthusiasm of nursing staff for learning relevant knowledge was not high. The management of patients with suspected COVID-19 symptoms has become a key step in prevention and control of the pandemic. Because many clinical symptoms caused by the new coronavirus are similar to those caused by influenza virus, parainfluenza virus, adenovirus, etc., the symptoms of COVID-19 are mainly atypical, with fever as the only typical symptom [15]. If health care professionals are not familiar with the processes to identify and manage patients with suspected COVID-19 symptoms, they are likely to fail to properly manage these patients in a timely manner, leading to further spread of the epidemic. Therefore, nursing staff must also further strengthen their training on the *Prevention and Control Plan for COVID-19* issued by the National Health Commission of China to improve the emergency response ability of nursing staff during this epidemic.

Infectious diseases are classified as public health emergencies and have the systematic characteristics of urgency, wide spread, complicated and changeable disease conditions, and serious harm. When an infectious disease epidemic occurs, it can easily cause anxiety, depression, and other epidemic-related stress reactions along with negative emotions in the public, including medical staff [16,17]. In this study, the TAF was used to evaluate the psychological status of the nursing staff who responded to the survey. The median score was 7.0, reflecting that the psychological damage suffered by the nursing staff was low. However, Lai et al [18] conducted a study involving 1830 heath care workers to assess the magnitude of mental health outcomes in Wuhan City, China. The results indicated that heath care workers, especially nurses, experienced more severe psychological burdens. One possible explanation for this difference from our study is the use of different investigation tools. However, among different personnel types, tenures, and departments, the difference in the scores of each dimension was statistically significant (*P*<.05). The main reason for this result is that nursing students during internship and nurses in standardized training have only just started clinical practice and lack rescue experience for disaster events, including public health emergencies [19,20]. Nursing staff with long tenure, meanwhile, have relatively high levels of psychological preparedness for disasters and already have experience in coping with epidemics, as they have experienced outbreaks of severe acute respiratory syndrome (SARS), avian influenza, H1N1 influenza, and other major infectious diseases. However, due to the extreme shortage of medical protective materials, especially protective clothing and masks, there is still a certain gap in the expectations of frontline departments with high exposure risks; therefore, the psychological damage among nursing staff in frontline departments is relatively high compared with that of nurses in the general department and other departments. It is suggested that all hospitals should strengthen the centralized and unified management of medical protection supplies, establish and use account books, accurately distribute materials, and carefully manage the situation to meet the needs of clinical workers.

### Limitations

This study has certain limitations. First, the content and structure of the knowledge questionnaire used in this study are relatively unique, and the reliability and validity of the structure have not been tested; this may affect the credibility of the results and lead to one-sidedness of the survey results. Second, the time period for investigating the psychological status of nursing staff was relatively short; therefore, the impact of the disease on the psychological health of the nursing staff may be underestimated, especially for those in job positions with high degrees of exposure. Finally, the investigation of the psychological status in this study mainly relies on a questionnaire and lacks an overall evaluation method, which may ignore potential influencing factors not included in the scale.

### Conclusion

The results of this study showed that nursing staff have insufficient knowledge about COVID-19 and should be retrained to strengthen their ability to manage and cope with the disease. Meanwhile, the nurse respondents showed minimal emotional, cognitive, behavioral, and overall psychological damage, and the impact of the disease on their psychological state was found to be relatively low at present. However, further study is required involving continuous follow-up observation combined with qualitative research to determine the mental health status of nursing staff in a timely manner and to provide a series of interventions.

## Abbreviations

COVID-19: coronavirus disease
SARS: severe acute respiratory syndrome
SARS-CoV: severe acute respiratory syndrome coronavirus
TAF: Triage Assessment Form
